# Radiation Exposure in Endovascular Surgery According to Complexity: Protocol for a Prospective Observational Study

**DOI:** 10.3390/mps6030049

**Published:** 2023-05-10

**Authors:** Dorelly Tanayra Martinez Del Carmen, Pablo Saldaña Gutierrez, Ramon Vila Coll, Elena Iborra Ortega

**Affiliations:** 1 Angiology and Vascular Surgery Department, Hospital Universitari de Bellvitge, 08907 L’Hospitalet de Llobregat, Barcelona, Spain; 2 Medical Physics and Radiological Protection Department, Institut Català d’Oncologia|Hospital Universitari de Bellvitge, 08907 L’Hospitalet de Llobregat, Barcelona, Spain

**Keywords:** endovascular procedures, interventional radiology, patient radiation protection, radiation dose, dosimetry, diagnostic reference level, angioplasty, endovascular aneurysm repair

## Abstract

In the past decades, we have witnessed tremendous developments in endovascular surgery. Nowadays, highly complex procedures are performed by minimally invasive means. A key point is equipment improvement. Modern C-arms provide advanced imaging capabilities, facilitating endovascular navigation with an adequate open surgical environment. Nevertheless, radiation exposure remains an issue of concern. This study aims to analyze radiation used during endovascular procedures according to complexity, comparing a mobile X-ray system with a hybrid room (fixed X-ray system). This is an observational and prospective study based on a cohort of non-randomized patients treated by endovascular procedures in a Vascular Surgery department using two imaging systems. The study is planned for a 3-year duration with a recruitment period of 30 months (beginning 20 July 2021) and a 1-month follow-up period for each patient. This is the first prospective study designed to describe the radiation dose according to the complexity of the procedure. Another strength of this study is that radiologic variables are obtained directly from the C-arm and no additional measurements are required for feasibility benefit. The results from this study will help us determine the level of radiation in different endovascular procedures, in view of their complexity.

## 1. Introduction

Treating patients with vascular pathology has experienced a revolution in recent years with the growth of endovascular procedures. This evolution is related to the advent of novel therapeutic tools (such as pressure balloons, drug balloons, stents, and endograft) and the advancement of X-ray technology.

We can perform this type of treatment using surgical X-ray equipment (also called C-arms). The term C-arm refers to equipment composed of a high-tension generator and a tube that produces X-rays, which penetrate the patient’s body, and converts these rays into a visible image [1].

Modern C-arms provide high-quality radiological images in real-time, enabling the execution of highly complex procedures that would be unthinkable with older equipment [2,3].

To perform these procedures in a sterile environment, we can use mobile C-arms in a standard operating room or fixed equipment in a so-called hybrid operating room, which combines the features of an operating room with a radiology room.

Nevertheless, radiation exposure and its potentially harmful effects on patients and healthcare professionals remain an issue of concern [2,3,4,5].

Increased exposure to ionizing radiation could lead to deterministic and stochastic injuries with immediate, intermediate, or long-term onset because of the increased number of diagnostic and therapeutic procedures performed on patients [1,2,3,4,5,6].

Deterministic effects (“tissue reactions”) are biological effects that occur as a result from the death of a large number of cells in a tissue or organ. They usually appear after a specific dose (threshold dose or threshold of clinical effects), being more severe the higher the amount received [1,4,5,6]. Skin lesions and lens opacities are examples of deterministic effects after radiation exposure [7,8,9,10].

Stochastic or probabilistic effects occur by chance, and their likelihood, but not severity, increases with increasing dose. There is no threshold dose, and effects can occur at low doses. The development of malignancy is the most common stochastic effect of radiation exposure [7,8,9,10]. Stochastic effects often appear years after exposure.

The decision to use a fixed C-arm or a mobile C-arm in our facility is made based on the availability of the X-ray equipment and the schedule of the procedures. In this study, we focus on arterial procedures: endovascular treatment of thoracic, abdominal, and thoracoabdominal aortic aneurysms, and procedures involving the revascularization of lower limbs.

Notwithstanding the widespread use of X-ray imaging units, there are a lack of published data about the impact of radiation exposure, and fixed C-arms are still related to higher radiation exposure [11,12,13].

The collection and subsequent analysis of radiological, clinical, and procedural parameters would identify opportunities for improvement in workflow and biosafety.

The results of this study may act as a springboard for further research on the comparison of radiation exposure between both X-ray equipment types according to complexity stratification of endovascular procedures. The identification of procedures that involve higher doses of radiation will allow the development of strategies to enhance safety in operating rooms where radiation exposure is involved.

## 2. Aims and Objectives

### 2.1. Primary Objective

This study aims to analyze radiation used during endovascular procedures according to complexity, comparing a mobile X-ray system (Philips Zenition 70 ^®^) with a hybrid room (Philips Azurion ^®^).

### 2.2. Secondary Objectives

The secondary objectives are: (1) Define the diagnostic reference radiation doses (DRLs) according to the procedure and its complexity, following the guidelines of the International Commission of Radiation Protection (ICRP) [14,15,16,17,18,19] and the European Council Directive 2013/59/EURATOM [20]. (2) Compare the dosimetric information provided in the dose reports of each procedure, based on the degree of complexity, in view of the implemented radiation protection measures. (3) Analyze the contrast dose used during the procedures on each device. (4) Associate the radiation dose of endovascular procedures with patients’ body mass index.

## 3. Methods

### 3.1. Study Design

This observational and prospective study is based on a cohort of non-randomized patients treated by endovascular procedures in a Vascular Surgery department.

### 3.2. Setting

The study is planned for 3-year duration with a recruitment period of 30 months and a 1-month follow-up period for each patient.

Patients will be recruited in outpatient consultations and hospitalization when the endovascular procedure is indicated according to standard clinical practice.

### 3.3. Participants

All patients who are candidates for an endovascular procedure will be invited to take part in the study. Oral and written information will be given.

### 3.4. Inclusion Criteria

All patients of ≥18 years agree to participate in the study by signing informed consent for one of the following procedures: (a) elective endovascular procedures for exclusion of thoracic and/or abdominal aortic aneurysm; (b) elective endovascular procedures for lower limb-threatening ischemia.

### 3.5. Exclusion Criteria

- Diagnostic arteriography and/or verification arteriography procedures;
- Endovascular procedures of upper limbs;
- Endovascular venous procedures.

### 3.6. Outcome Measures

Peak skin dose (PSD) is the primary outcome of this study. PSD is defined as the maximum absorbed dose to skin from exposure to X-rays [20]. Secondary outcomes are described in Table 1 [2,3,4,5,6,7].

### 3.7. Data Collection

The main source of information is the patient’s medical history and anamnesis. Radiological dose reports will be collected directly from the devices, including the main radiation dose metrics.

### 3.8. Data Variables

#### 3.8.1. Imaging Systems

The X-ray device used on each procedure will be described as a dichotomous variable: Mobile C-arm (MCA) or hybrid room (HR). The MCA equipment is Zenition 70^®^ (Philips, The Netherlands) with a 30 × 30 cm flat-panel detector. The C-arm is color-coded and fully balanced with ability to angulate from +90 to −50° [21].

Philips Azurion 7^®^ is the X-ray equipment in a HR with a 20” flat detector and equipped with a workstation for image processing and fusion imaging (VesselNavigator, Phillips Healthcare, Best, The Netherlands). All compatible applications in the interventional lab via the central touch screen module and FlexVision Pro [22].

#### 3.8.2. Endovascular Procedures

The type of procedures (Table 2), arterial approaches, and contrast volume will all be recorded [23,24].

#### 3.8.3. Complexity Levels

To obtain a reliable comparison across endovascular procedures, we have devised a framework for categorizing procedures on three levels of complexity considering type of surgery. In aortic procedures, the degree of complexity depends on arterial access, aortic neck anatomy, contralateral leg catheterization and iliac anatomy. For peripheral artery procedures we consider the Global Limb Anatomic Staging System (GLASS) (infrainguinal chronic limb threatening ischemia) [24], the Trans-Atlantic Inter-Society Consensus Document on Management of Peripheral Arterial Disease classification (TASC II) (aortoiliac lesion) [25], the possibility of recanalization, and the occurrence of dissection and/or thrombosis (Table 3).

### 3.9. Training and Professional Regulation

All team members are certified to perform endovascular treatment using X-ray imaging units and have been trained prior to enrollment to ensure adherence to monitoring and safety governance, focusing on the study dose protocol and the “As Low As Reasonably Achievable” (ALARA) principles.

Verifications of internal dosimeters readings of all systems are performed annually by the Medical Physics Department following Spanish National Regulation [18] and the Radiology Quality Assurance Protocol of the Spanish Medical Physics Association [19]. If a calibration is needed, the technical service is contacted.

## 4. Study Procedures

- Identify patients with scheduled endovascular procedures.
- Once the patient is scheduled for endovascular surgery, we will explain the study and request their participation. If the patient agrees to participate, they will sign an informed consent document.
- The procedure will be scheduled either in the hybrid room or in the conventional room with a mobile C-arm, according to their availability.
- Collect information about medical history and anthropometric data during the interview and from the medical records in a database.
- The scheduled endovascular procedure will be carried out according to standard clinical practice.
- Obtain radiation dose parameters extracted directly from Philips Azurion^®^ and Philips Zenition 70^®^.
- If the endovascular procedure exceeds the radiation dose threshold (DAP ≥ 500 Gy·cm^2^, PSD ≥ 2000 mGy or AK ≥ 2000 mGy), the coordinating researcher will receive an alarm and will perform a patient clinical evaluation looking for any warning symptoms or signs.
  - In case of not finding signs of radiodermatitis, the patient will be informed of the warning signs, we will provide an information sheet, and preferential outpatient check-ups will be scheduled.
  - In case of presenting clinical signs of radiodermatitis, the patient will be evaluated by a dermatologist.
- Based on standard clinical practice, patients will be assessed at one month.

## 5. Statistical Analysis

### 5.1. Sample Size Calculation

This research focuses on endovascular operations carried out in a high surgical volume. The number of procedures performed throughout the recruitment period will determine the sample size, which is anticipated to be between 150 and 200 endovascular procedures annually.

### 5.2. Statistical Analysis

We will perform a general descriptive analysis of the study variables. The results will be expressed as means and standard deviation, median, maximum, and minimum value, range and third quartile for the quantitative variables and for the categorical variables, the absolute and relative frequencies of each category. All study variables will be described, and descriptive statistics will be used according to the nature of the variable.

The principal analysis is the peak skin dose and other radiation dose variables described in Table 1. It will be analyzed by indirect radiological parameters, such as KAP or PSD. The secondary research will include:

− The study population will be described according to the baseline characteristics and the type of procedure.
− The procedure performed according to X-ray device used (mobile vs. fixed) and the level of complexity of the procedure will be described.
− Partnership studies will be carried out.

Estimators will be accompanied by a 95% confidence interval. Statistical significance has been set at a probability level of <0.05.

## 6. Data Management and Quality Control

During the study period, we will collect data from electronic medical records. All patients undergoing endovascular procedures during the study period will be selected and included if they meet the inclusion criteria. All the data obtained will be anonymized and a consecutive participation number will be assigned as the inclusion process is executed. Once this process has been accomplished, statistical analysis will be performed. These activities will be supervised by the coordinating researcher of the study.

## 7. Patient and Public Involvement

Patients and/or the public were not involved in the design, conduct, reporting, or dissemination plans of this research.

## 8. Ethics, Approval and Dissemination

The treatment of patient data within the framework of this study will comply with the provisions of current legal regulations at the national and European level: Organic Law 3/2018 on the Protection of Personal Data and Guarantee of Digital Rights, and Regulation (EU) 2016/679 General Data Protection. Patient data will be coded using a numerical code for the purposes of this study, eliminating any identifying or identifiable data. The research file will be kept in an electronic directory of the center, with restricted access, under the supervision of Information Systems of the Hospital Universitari de Bellvitge.

The researcher agrees to disseminate and publish the results in accordance with current legislation and the Declaration of Helsinki (Fortaleza, 2013). In the publications and other means of disseminating the results, the anonymity of the patients will be maintained.

Any written or oral communication of the research results must be approved by the research coordinating investigator and, where appropriate, in collaboration with the associated scientists.

All data transferred between sites will be encrypted, and no individual will be identifiable from the stored data. Identifiable patient information will be stored in a locked cabinet, accessible only by research members at the site of the data collection. The clean database file will be anonymized and kept for 25 years.

## 9. Discussion

X-ray exposure has been studied in recent years, especially during endovascular exclusion of abdominal aortic aneurysms [24,25,26]. Of particular interest is the comparison between the doses used depending on the equipment used: fixed or mobile. The scarcity of studies that analyze radiation based on the type of procedure and its complexity persists. For all these reasons, we believe that the present study can contribute knowledge in little-explored areas, such as the analysis of the radiation dose considering the level of complexity of the procedures on endovascular surgery.

The increasing number of endovascular procedures performed nowadays determines a greater exposure to ionizing radiation by patients and professionals during diagnosis, treatment, or follow-up. X-rays and their possible harmful effect remain subjects of study. With this study, we plan to analyze the radiation dose during the endovascular procedures performed in a Vascular Surgery department.

Radiation dose monitoring in endovascular surgery allows the analysis of X-ray exposure of patients and healthcare professionals involved. Additionally, strategies could be developed to lower the absorbed dose and the yearly number of procedures with high levels of dose area product and air kerma [8,22,26].

## 10. Conclusions

In conclusion, the analysis of radiation data of endovascular procedures is essential to detect which imaging systems and procedures involve higher doses. This is an observational and prospective study that attempts to examine the radiation dosage in two distinct imaging systems, using a classification of complexity levels in order to achieve a reliable comparison.

## Figures and Tables

**Table 1:** Description of other radiation dose measures.

Parameters	Description
**Air kerma (AK)**	Represents the kinetic energy released per unit mass when an X-ray beam travels through air. The unit is milliGrays (mGy). It will be recorded by the system.
**Air-kerma area product (KAP or dose area product, DAP)**	The KAP is the product of two factors, namely the air kerma free in the air over the area of the X-ray beam in a plane perpendicular to the beam axis.
**Fluoroscopy time (FT)**	The time of exposure to X-ray, expressed in seconds. Fluoroscopy is a radiographic technique used for the visualization of anatomical structures in motion that allows guiding different diagnostic and therapeutic procedures in real-time. The unit is seconds. It will be recorded by the system.
**Contrast volume**	The total volume of contrast injected into the patient during the procedure. The unit is milliliter (mL).
**Body mass index (BMI)**	The measure of the weight compared to the height. The total amount of dose delivered will be correlated with patient size and weight.
**Number of DSA series**	The total number of digital subtraction series.
**Procedure time**	The total time interval from the start and end of the procedures, recorded in minutes (min).
**Type of anesthesia**	Described as general or loco-regional.

**Table 2:** List of endovascular procedures.

Endovascular Procedures	Description
(1) Endovascular exclusion of abdominal aortic aneurysm	Placement of stent graft for internal lining of the aorta to exclude abdominal aneurysm.
(2) Endovascular exclusion of thoracic aorta	Placement of stent graft for internal lining of the thoracic aorta to exclude aneurysm, dissection, intramural hematoma, penetrating aortic ulcer or traumatic aortic injury.
(3) Endovascular exclusion of thoracoabdominal aortic aneurysm	Placement of stent graft for internal lining of the aorta to exclude thoraco-abdominal aneurysm.
(4) Endoleaks repair	Procedures that involve repairing blood flow between the graft and the aneurysm.
(5) Iliac percutaneous transluminal angioplasty or recanalization	Flow restoration procedure of the iliac sector through dilation with balloon catheter, with or without stent placement.
(6) Iliac stent	Stent placement in the iliac sector.
(7) Femoropopliteal percutaneous transluminal angioplasty or recanalization	Flow restoration procedure of femoropopliteal sector with or without stent placement.
(8) Femoropopliteal stent	Stent placement in the femoropopliteal sector.
(9) Infrapopliteal percutaneous transluminal angioplasty	Flow restoration procedure of the infrapopliteal sector through dilation with balloon catheter.

**Table 3:** Complexity levels of endovascular procedures.

Low Complexity
A. Angioplasty of any territory
1. Arterial puncture without incidents 2. The guide passes easily without requiring specific material changes or multiple attempts 3. Absence of complication in the final angiogram 4. No arterial access complications
**Medium Complexity** (if any of the following are true)
A. Angioplasty of any territory
1. Difficult arterial puncture but achievable 2. Contralateral approach 3. Laborious recanalization of the stenosis/occlusion requiring one of the following conditions: guidewire/catheter changes collaboration of a second vascular surgeon 4. Presence (or not) of residual dissection on final angiogram 5. Presence (or not) of complications of arterial access
B. Angioplasty and stenting of any territory: whenever a stent implant is required, it will be of medium complexity (at least)—TASC A/B (aortoiliac), GLASS 1–2.
C. Recanalization of any territory—TASC A/B (aortoiliac)
D. Aortic endoprosthesis
1. An arterial approach without incidents (open or percutaneous) 2. Non-hostile anatomy (neck length > 15 mm and/or neck diameter < 32 mm, length of iliac arteries > 15 mm and/or diameter <20 mm) 3. Implantation of the device without incident 4. Easy catheterization of the contralateral limb 5. Absence of endoleaks in the final angiographic control (except type II)
**High Complexity**
A. Angioplasty/stent of any territory
1. Difficult arterial puncture requiring a change to open approach or arterialficult arterial puncture requiring a change to open approach or arterial access 2. Access from upper limb (UL) 3. Laborious recanalization of the stenosis/occlusion requiring one of the following conditions: guidewire/catheter changes collaboration of a second vascular surgeon retrograde puncture 4. Final angiogram with dissection/thrombosis requiring extra procedures (for example thrombectomy) 5. Presence of complications of arterial access
B. Recanalization of any territory
1. Difficult puncture requiring a change of approach point/open approach 2. Access from UL 3. Recanalization of an entire arterial axis (iliac, femoral and/or distal trunks)—TASC C/D (aorto-iliac), GLASS 3. 4. Laborious recanalization of the stenosis/occlusion requiring one of the following conditions: guidewire/catheter changes collaboration of a second vascular surgeon retrograde puncture 5. Final angiogram with dissection/thrombosis requiring extra procedures (for example thrombectomy) 6. Presence of complications of arterial access 7. Despite multiple attempts, it is not possible to recanalize the occlusion
C. Aortic endoprosthesis
1. Complication of the arterial access (open or percutaneous) 2. Hostile anatomy (neck length < 15 mm and/or neck diameter > 32 mm, length of iliac arteries < 15 mm and/or diameter > 20 mm) 3. Complex catheterization of the contralateral limb requiring extra procedures (for example, single-loop snare) 4. Additional procedures for endoleak treatment (such as central cuff extension or additional iliac limb) 5. Additional procedures as embolization of inferior mesenteric/internal iliac artery, placement of endo-anchor, etc. 6. Chimney or snorkel technique 7. Fenestrated or branched aortic endograft 8. Iliac branch

## Data Availability

Not applicable at the time of publication. The dataset used and/or analyzed during the current study will be available from the corresponding author upon reasonable request.
